# Insecticidal and Acetylcholinesterase Inhibition Activity of *Veratrum nigrum* Alkaloidal Extract against the German Cockroach (*Blattella germanica*)

**Published:** 2018-12-25

**Authors:** Xianghai Cai, Qingfeng Li, Lei Xiao, Hailiang Lu, Jian Tang, Jianbo Huang, Jianzhong Yuan

**Affiliations:** 1 State Key Laboratory of Phytochemistry and Plant Resources in West China, Kunming Institute of Botany, Chinese Academy of Sciences, China; 2 Institute of Plant Physiology and Ecology, Shanghai Institutes for Biological Sciences, Chinese Academy of Sciences, Shanghai, China; 3 Infinitus (China) Limited Company, Guangzhou, China

**Keywords:** Insecticidal activity, Acetylcholinesterase inhibition, *Veratrum nigrum*, Medicinal plant, German cockroach

## Abstract

**Background:**

*Veratrum nigrum* (Liliaceae) is perennial medicinal plant widely used to treat various conditions. To determine its insecticidal properties against the German cockroach (*Blattella germanica*), several laboratory tests were carried out.

**Methods:**

A 4kg dry sample of *V. nigrum* root was purchased from the medicinal material market in Yunnan Province in 2015, China. In contact toxicity tests, *V. nigrum* alkaloidal extract was topically applied to the abdomen of cockroaches using a micro-applicator. In vitro acetylcholinesterase (AChE) activity tests were performed using a modified Ellman method.

**Results:**

*Veratrum nigrum* alkaloidal extract was toxic to male adults and 4^th^ nymphs cockroaches, with median lethal dose (LD_50_) values of 14.90μg/insect, 14.21μg/insect for adults and 41.45μg/insect, 39.01μg/insect for 4^th^ nymphs after 24h and 48h exposure, respectively. There was a significant difference between adults and nymphs in terms of tolerance to *V. nigrum* alkaloidal extract. There was no significant difference in mortalities at 24h and 48h, the lethal effect of *V. nigrum* alkaloidal extract on German cockroach was quick. AChE activity tests showed that *V. nigrum* alkaloidal extract had an excellent inhibitory effect on AChE: inhibition in the 4^th^ nymphs and male adults had 50% inhibiting concentration (IC_50_) values of 3.56mg/ml and 5.78mg/ml respectively. The inhibitory effect of AChE activity was positively correlated with inhibitory time (0–20min), at a concentration of 1mg/ml, inhibition of nymph and adult AChE activity had 50% inhibiting time (IT_50_) values of 8.34min and 16.75min, respectively.

**Conclusion:**

*V. nigrum* may be explored as a potential natural insecticide for control of the German cockroach.

## Introduction

The German cockroach, *Blattella germanica* Linnaeus is both a serious pest and a mechanical vector for bacteria and other pathogens (1–3). To date, the control of cockroaches in China mainly has relied on two types of insecticides administered as sprays or toxic baits based on synthetic insecticides. Toxic baits may be more effective than spray formulations against German cockroaches (4). Synthetic insecticides are an effective tool to manage pests but are associated with negative effects such as insecticide resistance, environmental pollution and human health problems. Over 500 insect species had developed resistance (5). The resistance of the German cockroach to different insecticides is also very serious (6, 7). Therefore, it is essential to develop a new type of environmentally-friendly insecticide. Plant extracts are undoubtedly an ideal candidate due to their low mammalian toxicity, readily biodegradable and low risk to the environment (8).

Many medicinal plant extracts have been reported to show insecticidal properties against public health pests, such as mosquitoes (9, 10), houseflies (11, 12) and German cockroaches (*B. germanica*) (13, 14). One such example is *Veratrum nigrum* Linnaeus which belongs to the Liliaceae family. It is a perennial traditional Chinese medical plant and is very prevalent in the wild in China. It has been extensively used for the treatment of hypertension, stroke, excessive phlegm and epilepsy (15, 16). In recent years, it had other properties, such as antifungal (15) and insecticidal activity (17, 18). However, there are few studies on the insecticidal activity of *V. nigrum* against public health pests such as the *B. germanica*. Therefore, we investigated the insecticidal activity of *V. nigrum* against *B. germanica*.

## Materials and Methods

### Preparation of *V. nigrum* alkaloidal extract

A 4kg dry sample of *V. nigrum* root was purchased from the medicinal material market in Yunnan Province in 2015, China. The sample was crushed and extracted three times with 15L methanol at room temperature. The extraction liquid was made up to 1000ml with water after removal in vacuo*.* The extract was partitioned between 1% hydrochloric acid solution and ethyl acetate. Components of the acidic layer, adjusted to pH 9–10 with 10% ammonia solution, were extracted with ethyl acetate to give an alkaloidal extract (69g).

### Compounds

Acetylthiocholine iodide, 5, 5’-dithiobis (2-nitrobenzoic acid) (DTNB), Triton X-100, and eserine were purchased from Sigma Chemical Company. Positive control, tetramethrin (95%) was purchased from Shanghai Forever Chemical Co, Ltd.

### Insect rearing

*Blattella germanica* strain maintained at 26±1 °C and 60% relative humidity under 16:8h (L:D) cycle in the laboratory for 40yr without exposure to any insecticides.

### Contact toxicity test

A group of 20 male adult or 4^th^ nymph cockroaches were anesthetized using Ether. A droplet (1μl) containing five different concentrations of *V. nigrum* alkaloidal extract (0.375–12%) or tetramethrin (0.375–6%) was topically applied to the abdomen of cockroaches using a micro-applicator (Burkard, UK). A 1μl droplet of acetone was applied to the control group. After treatment, cockroaches were transferred to a 200ml glass bottle maintained at a temperature of 26±1 °C and a humidity of 75±10% RH. Mortality was assessed 24h and 48h after treatment. All experiments were repeated in triplicate.

### In vitro acetylcholinesterase (AChE) activity test

In vitro acetylcholinesterase (AChE) activity tests were performed as follows: 10 male adults and 4^th^ nymph cockroaches were homogenized using a glass tissue grinder in ice in 20mM phosphate buffer (pH 7.0) containing 0.5% Triton X-100. The homogenate was then centrifuged at 12000gr for 15min at 4 °C. The supernatant was used as an enzyme source for measuring AChE activity via a modified Ellman method (19). To determine the 50% inhibiting concentration (IC_50_) of the alkaloidal extract, 50μl of the enzyme was added to a test tube containing 50μl of six different concentrations (0.375–12 mg/ml) of the alkaloidal extract and pre-incubated at 25 °C for 5min. To determine the 50% inhibiting time (IT_50_), the above procedure was followed and pre-incubated at 25 °C for six different time periods (0.5min, 1min, 5min, 10min, 15min, 20min). After incubation, 1300μl of 20 mM Ellman solution containing 1mM acetylthiocholine iodide, 0.23mM 5′, 5′-dithiobis (2-nitrobenzoic acid) and 0.45mM NaHCO_3_ were added to the test tube which was then incubated at 25 °C for 20min. The reaction was stopped by 100μl 10mM eserine and the absorbance at 412nm was measured. Each assay was replicated five times.

### Statistical analysis

Mortality corrected using Abbott’s formula. All analyses were performed using SPSS 13.0 software (Chicago, IL, USA). LD_50_, IC_50_ and IT_50_ were considered to be significantly different on the basis of non-overlapping of 95% confidence intervals (CLs).

## Results

### Contact toxicity test

Contact toxicities of *V. nigrum* alkaloidal extract against male adults and 4^th^ nymphs cockroaches are shown in Table 1. *Veratrum nigrum* alkaloidal extract has significant insecticidal activity against the *B. germanica*. There was no significant difference in mortality at 24h and 48h (overlapping of 95% CL) at LD_50_ values of 14.90μg/insect, 14.21μg/insect for adults and 41.45μg/insect, 39.01μg/insect for 4^th^ nymphs after 24h and 48h exposure, respectively. The lethal effect of *V. nigrum* alkaloidal extract on *B. germanica* is quick.

**Table 1.** Toxicity of *Veratrum nigrum* alkaloidal extract against male adults and 4^th^ nymphs of *Blattella germanica* after exposure for 24 and 48h

**Compounds (μg/insect)**	**Age**	**Exposure time**	**LD_50_±SD**	**95%fiducial limits**	**Slope±SD**	**Chi square (χ^2^)**
**Alkaloidal extract**	Adult	24	14.90±1.35	13.40–16.68	3.33±0.42	1.99
	4^th^ Nymph	24	41.45±4.45	36.02–47.68	2.27±0.33	3.73
**Tetramethrin**	Adult	24	7.89±0.88	6.89–9.93	3.39±0.40	2.89
	4^th^ Nymph	24	13.98±1.26	10.82–18.22	2.79±0.38	2.43
**Alkaloidal extract**	Adult	48	14.21±1.40	13.12–15.78	3.23±0.38	2.25
	4^th^ Nymph	48	39.01±4.03	35.55–46.89	2.65±0.37	3.68
**Tetramethrin**	Adult	48	7.72±0.78	6.52–8.90	3.21±0.37	2.76
	4^th^ Nymph	48	13.30±1.28	10.32–18.00	2.81±0.33	2.41

### In vitro acetylcholinesterase (AChE) activity test

**Fig. 1.**
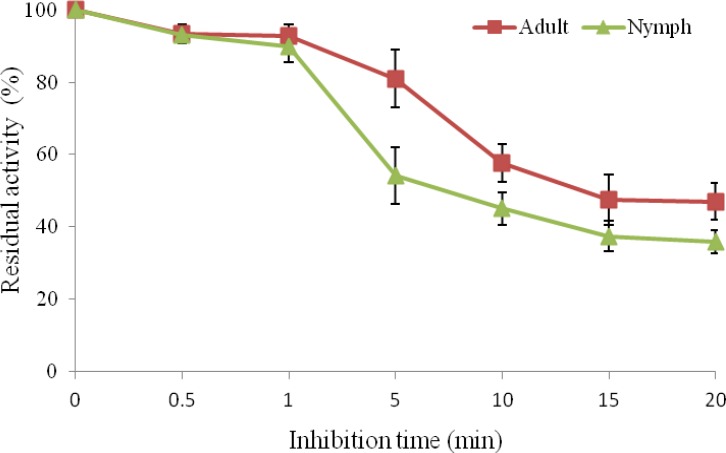
Residual AChE activity in male adults and 4^th^ nymphs of *Blattella germanica* at 1 mg/ml *Veratrum nigrum* extract

The results of in vitro acetylcholinesterase (AChE) activity tests are shown in Table 2 and Fig. 1. *Veratrum nigrum* alkaloidal extract was found to have an excellent inhibitory effect on AChE, showing IC_50_ values were 3.56mg/ml for nymphs and 5.78mg/ml for adults, respectively (Table 2). Inhibition of AChE activity was positively correlated with inhibition time. When the concentration of *V. nigrum* alkaloidal extract was 1mg/ml, inhibition of AChE activity in both nymphs and adults was at IT_50_ values of 8.34 min and 16.75min, respectively (Fig. 1).

**Table 2.** IC_50_ values of *Veratrum nigrum* alkaloidal extract against male adults and 4^th^ nymphs of *Blattella germanica* AChE activity

**Age**	**IC_50_ (mg/ml) (±SD)**	**95% Confidence limit**	**Slope (±SD)**	**Chi square (χ^2^)**
**Adult**	5.78±0.47	4.78–6.98	1.24±0.13	1.54
**4^th^ nymph**	3.56±0.49	2.36–4.72	1.35±0.11	1.35

## Discussion

The Chinese pharmacopoeia includes traditional Chinese medicine plants, shown to have anti-oxidant, free radical scavenging, anti-inflammatory and anticancer properties (20). In previous studies, *V. nigrum* extracts had insecticidal effects against *Mythimna separata*, *Aphis craccivora*, *Cx. pipiens pallens*, *Tetranychus cinnabarinus*, and *Plutella xylostella* (17, 18). Certain Chinese medicinal plant extracts have been reported to be bioactive against *B. germanica*, such as *Pogostemon cablin* at a LD_50_ of 23.45 μg/insect (14), and *Chenopodium ambrosioides* at a LD_50_ of 64.47μg/insect (13).

Our results showed that *V. nigrum* alkaloidal extract has significant insecticidal activity against *B. germanica*. Compared with the control (a chemical insecticide: tetrarmethrin, LD_50_ of 7.89 μg/insect, 7.72μg/insect for adults and 13.98μg/insect, 13.30μg/insect for 4^th^ nymphs after 24h and 48h exposure, respectively), *V. nigrum* alkaloidal extract showed a little weaker or equivalent toxic to the *B. germanica*. Contact toxicity of *V. nigrum* alkaloidal extract against male adults was stronger than 4^th^ nymphs *B. germanica*. Moreover, the result was similar to insecticidal activities of tetramethrin against above both. The tolerance level of the nymphs was significantly higher than that of adults (non-overlapping of 95% CL), as also reported by (21–23). The weight difference between males and females may be one reason for the different susceptibility to insecticide (24, 25). In our research, the mean nymph weight (30.18 mg) was lighter than the adult male (54.33mg) but did not appear to be a factor for the increase intolerance. Therefore, there may be other modes of action responsible for this difference, but the mechanisms of plant extract toxicity against *B. germanica* are poorly reported.

The insecticidal target site of the phytochemicals against cockroaches has been previously researched, such as the insect GABA receptor was target site for carvacrol, pulegone, and thymol (26). Octopamine receptor was target site for carvacrol, α-terpineol and pulegone (27, 28). In addition, the AChE inhibition activity of phytochemicals on *B. germanica* has been previously studied in vitro (24, 29, 30). In the present study, *V. nigrum* had an excellent inhibitory effect on AChE, showing IC_50_ and IT_50_ values were 3.56mg/ml, 8.34min for nymphs and 5.78mg/ml, 16.75min for adults, respectively. AChE in 4^th^ nymph *B. germanica* was more susceptible to insecticide than in male adult cockroaches, similar to results found by (24, 29).

The mechanisms of insecticide resistance in *B. germanica* have many factors, including target site insensitivity, metabolic detoxification and decreased skin permeability (31, 32). Therefore it is difficult to explain the higher tolerance of nymphs against *V. nigrum* alkaloidal extract through AChE inhibition alone.

In addition, plant extracts have many active ingredients and biological activity is, therefore, the result of a combination of various components (13, 33). It is necessary to determine the active components of *V. nigrum* which are currently poorly understood for insecticidal efficacy. In recent years, 35 active ingredients were identified in *V. nigrum* alkaloidal extract and the main components were veratramine, followed by veratrosine and jervine (34). This would provide a strong foundation for screening the insecticidal constituents of *V. nigrum*. Further work focuses on insecticidal activities of *V. nigrum* ingredients against *B. germanica*.

## Conclusion

Insecticidal activities of phytochemicals derived from Chinese medicinal plants have been previously investigated against *B. germanica*. *V. nigrum* (the family of Liliaceae) alkaloidal extract have excellent insecticidal activity compared to tetramethrin in vivo and inhibitory effect on AChE in vitro*.* It showed potential for further development as possible natural insecticide against *B. germanica*. However, further research is also necessary, such as the activities of ingredients and the modes of action of the individual ingredient.
